# Hyperhomocysteinemia and Endothelial Dysfunction in Multiple Sclerosis

**DOI:** 10.3390/brainsci10090637

**Published:** 2020-09-16

**Authors:** Ekaterina Dubchenko, Alexander Ivanov, Natalia Spirina, Nina Smirnova, Mikhail Melnikov, Alexey Boyko, Evgeniy Gusev, Aslan Kubatiev

**Affiliations:** 1 Department of Neuroimmunology of Federal Center of Brain and Neurotechnology of the Federal Medical-Biological Agency of Russia, 117997 Moscow, Russia; katya.dubchenko@gmail.com (E.D.); n.f.smirnova@rambler.ru (N.S.); boykoan13@gmail.com (A.B.); 2 Interdistrict Department of Multiple Sclerosis at the State Clinical Hospital VV Veresaeva, 127644 Moscow, Russia; 3 Federal State Budgetary Scientific Institution “Institute of General Pathology and Pathophysiology”, 125315 Moscow, Russia; ivanov_av82@mail.ru (A.I.); niiopp@mail.ru (A.K.); 4 Department of Nervous Diseases with Medical Genetics and Neurosurgery Yaroslavl State Medical University, 150000 Yaroslavl, Russia; nnspirin@yandex.ru; 5 Department of Neurology, Neurosurgery and Medical Genetic of Pirogov Russian National Research Medical University, 117997 Moscow, Russia; gusevei@yandex.ru; 6 Laboratory of Clinical Immunology, National Research Center Institute of Immunology of the Federal Medical-Biological Agency of Russia, 115478 Moscow, Russia

**Keywords:** multiple sclerosis, endothelial dysfunction, homocysteine, hyperhomocysteinemia

## Abstract

Endothelial dysfunction is recognized as one of the leading factors in the pathogenesis of diseases of the central nervous system of various etiologies. Numerous studies have shown the role of hyperhomocysteinemia in the development of endothelial dysfunction and the prothrombogenic state. The most important condition in the development of multiple sclerosis (MS) is a dysregulation of the blood-brain barrier (BBB) and transendothelial leukocyte migration. It has been proven that homocysteine also contributes to the damage of neurons by the mechanism of excitotoxicity and the induction of the apoptosis of neurons. These processes can be one of the factors of neurodegenerative brain damage, which plays a leading role in the progression of MS. This review describes the pleiotropic effect of homocysteine on these processes and its role in MS pathogenesis.

## 1. Introduction

Endothelial dysfunction is currently considered as one of the universal mechanisms for the development and progression of damage to the nervous system in diseases of various etiologies. A large number of experimental and clinical studies have convincingly shown that the development of endothelial dysfunction is an important factor in the pathogenesis of not only vascular but also autoimmune and neurodegenerative diseases [1,2]. This review describes the pleiotropic effect of homocysteine on these processes and its role in multiple sclerosis (MS) pathogenesis.

## 2. Endothelial Dysfunction in Multiple Sclerosis

The term “endothelial dysfunction” means a change in the functions of the endothelium, accompanied by a decrease in the formation of a number of vasodilators (nitric oxide, prostacyclins, and others) with the formation of a proinflammatory and prothrombotic state [3,4,5]. The role of endothelial dysfunction in the development and progression of cardiovascular pathology, including acute and chronic forms of cerebrovascular diseases, has been most studied. The development of endothelial dysfunction in cerebrovascular pathology is associated with the impact of risk factors (hyperlipidemia, arterial hypertension, and others) and is accompanied by a number of changes. It has been shown that the development of endothelial dysfunction is accompanied by an increase in the expression of cell adhesion molecules, an increase in the activity and aggregation of platelets, an increase in the penetration of low-density lipoproteins into the intima, as well as an increase in oxidative stress. The combination of these changes creates conditions conducive to atherogenesis [4].

In recent years, the role of endothelial dysfunction in multiple sclerosis (MS) pathogenesis has been actively studied. The interest in studying endothelial function in MS is due to the fact that the dysregulation of the blood-brain barrier (BBB) and the transendothelial migration of leukocytes is one of the first and most important links in the pathogenesis of MS [6,7,8,9,10,11]. Damage to the BBB in MS is probably closely associated with impaired endothelial function under the influence of proinflammatory cytokines and a decrease in the synthesis of endothelial binding proteins. MS is characterized by an increase in the expression of cell adhesion molecules (Vascular Cell Adhesion Molecule 1 (VCAM-1), Intercellular Adhesion Molecule 1 (ICAM-1)) on the surface of endotheliocytes, which is an important factor ensuring the penetration of activated leukocytes through the BBB [11]. The increased expression of cell adhesion molecules may be one of the main factors in the formation of plaques characteristic of MS, which are localized mainly in the immediate vicinity of small vessels, mainly venules [12]. Generalized damage to the vascular endothelium of the central nervous system in MS was also confirmed in pathomorphological studies, which revealed dystrophic changes in endotheliocytes and pericytes of the microvasculature, the thinning and discontinuity of the inner and outer basement membranes, as well as pathological changes in the venule wall [13]. The role of the vascular factor in the pathogenesis of MS is also indicated by studies using modern neuroimaging methods that allow the detection of cerebral hypoperfusion [14]. In our study [15], using single-photon emission computed tomography (SPECT) showed a significant decrease in perfusion in MS patients over 45 years old, characteristic of vascular brain damage. The detection of an enlarged venule in the center of the demyelination focus in MS, detected by MRI, is now proposed as a key differential diagnostic criterion for MS [16].

In addition, in patients with MS, the laboratory signs of endothelial dysfunction were revealed—increased levels of von Willebrand factor antigen and adhesion molecules (sICAM-1, soluble platelet endothelial cell adhesion molecule 1 (sPECAM-1), sE-selectin, sP-selectin) as well as desquamated endothelial cells in comparison with healthy volunteers, which increase with an increase in the activity of the disease (the development of an exacerbation). These data indicate an important role of endothelial damage in the pathogenesis of MS [17].

## 3. The Role of Homocysteine in Endothelial Dysfunction in Multiple Sclerosis

**Figure 1:**
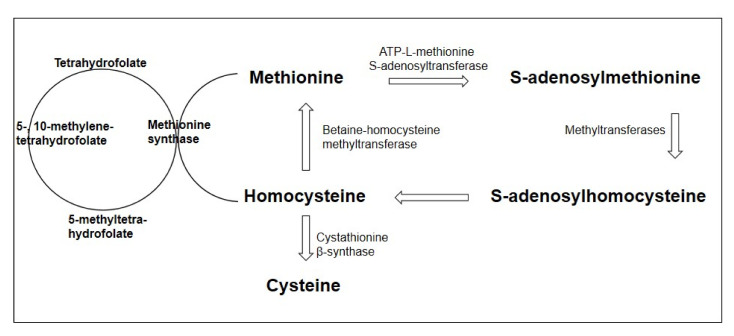
Homocysteine metabolism pathway (adapted from Esse et al., 2019 [19]).

One of the crucial factors in the development of endothelial dysfunction in various conditions is an increase in the content of homocysteine in the blood plasma, a sulfur-containing acid that is a product of methionine metabolism. Homocysteine metabolism is mediated by two main pathways: transsulfuration to cysteine and remethylation to methionine. The key enzymes for providing these metabolic pathways are cystathionine-β-synthetase (CBS) and methylenetetrahydrofolate reductase (MTHFR), respectively. Homocysteine metabolism is primarily determined by the content of folates, vitamin B12, and vitamin B6, which is a coenzyme in the demethylation reaction of homocysteine, which acts as a cofactor for remethylation [18]. The homocysteine metabolism pathway is presented in Figure 1.

Hyperhomocysteinemia is defined as an increase in the plasma homocysteine concentration above 10 μM/L. The main reasons for the development of hyperhomocysteinemia are mutations in the genes encoding homocysteine metabolism enzymes; a deficiency of cofactors for the reactions of homocysteine metabolism—folic acid, vitamins B12 and B6; chronic renal failure; alcoholism; hypothyroidism; and taking a number of medications [20].

Currently, hyperhomocysteinemia is considered as one of the most important modifiable risk factors for cardiovascular diseases [20]. According to a meta-analysis of a series of prospective studies, an increase in plasma homocysteine levels by 25% is associated with an increase in stroke risk by 19% and myocardial infarction by 11% [21].

Currently, the presence of several main mechanisms of the negative influence of homocysteine has been shown. Homocysteine, in contrast to other aminothiols (cysteine, glutathione, N-acetylcysteine), causes oxidative stress by acting through various mechanisms (direct and indirect), the role of which have not yet been adequately studied. This, in turn, leads to the development of endothelial dysfunction and the formation of a prothrombogenic state. In cells, a small part of homocysteine is converted into the thiolactone form, which easily penetrates through membranes and has a high biological activity. Thus, it was shown that thiolactone homocysteine inhibits Na^+^/K^+^-ATPase [22] and lysyl oxidase [23]. However, the role of these mechanisms in the development of endothelial dysfunction is unclear. Although homocysteine is not included in the primary structure of proteins, it can modify their cysteine and lysine residues, disrupting the functions of proteins, and imparting antigenic properties to them [24]; however, there are no data on the role of protein homocysteinylation in the development of autoimmune response in MS. The role of hypomethylation in the development of endothelial dysfunction in conditions of hyperhomocysteinemia has been more studied [25]. With an increase in the intracellular concentration of homocysteine, the decomposition reaction of its precursor, S-adenosyl homocysteine (SAH), is inhibited. An excess of SAH, in turn, inhibits the transmethylation reactions of proteins, DNA, and other substrates, which leads to multiple changes in gene expression. Hypomethylation, in turn, inhibits the expression of cystathionine-γ-lyase, an enzyme of the transsulfuration pathway that is involved in the removal of excess homocysteine and the synthesis of the H2S vasodilator [26,27]. It is now believed that hypomethylation plays a key role in homocysteine-mediated proliferation. In addition, homocysteine stimulates the proliferation of smooth muscle cells in the vascular wall [28,29,30].

Hyperhomocysteinemia is associated with an increased risk of acute cerebrovascular accidents in ischemic and hemorrhagic types due to the promotion of the atherogenesis and atherothrombosis of cerebral vessels increase in the activity of matrix metalloproteinases [30,31]. Hyperhomocysteinemia is also of great importance in chronic forms of cerebrovascular pathology [29,32]. It has been shown that the presence of hyperhomocysteinemia is an independent risk factor for the development of cognitive impairment [33,34,35], and the concentration of homocysteine in blood plasma statistically significantly negatively correlates with cognitive functions. At the same time, this relationship is more pronounced in the cognitive impairment of vascular etiology [29]. On the other hand, clinical studies on the effect of decreased homocysteine levels on cognitive function in the elderly have shown conflicting results [29].

Currently, the study of the role of homocysteine in a number of autoimmune and neurodegenerative diseases attracts much attention from researchers [36]. In patients with MS, a number of studies have revealed a statistically significant increase in homocysteine concentration compared to healthy volunteers [37,38,39,40].

In addition to the aforementioned ability of homocysteine to induce oxidative stress, it also causes neuronal damage through the mechanism of excitotoxicity and the induction of neuronal apoptosis [36,41,42,43]. Homocysteine is an agonist of both subtypes of glutamate receptors (α-amino-3-hydroxy-5-methyl-4-isoxazolepropionic acid receptor (AMPA) and N-methyl-D-aspartate receptor (NMDA)), the stimulation of which leads to an increase in the intracellular calcium concentration, an increase in ROS production, and the activation of caspases. In addition, it has been shown that homocysteine has a toxic effect on glial cells [44] Another possible aspect of the negative effect of homocysteine in MS may be the hypomethylation of myelin basic protein, which develops due to a decrease in the availability of S-adenosylmethionine in hyperhomocysteinemia, which leads to the destabilization of the myelin structure [45,46]. Importantly, in this meta-analysis hyperhomocysteinemia in MS is not accompanied by statistically significant changes in the folate and vitamin B12 levels [46].

Thus, the direct neurotoxic effect of homocysteine, which is realized through the mechanisms of excitotoxic damage to neurons, may be one of the factors of neurodegenerative brain damage that plays a decisive role in MS progression [36,47,48]. The most convincing data on the association of hyperhomocysteinemia with MS progression came from the study by Teunissen et al. [49], which showed that the homocysteine concentration correlates with the clinical progression of the disease according to the prospective observation of patients. According to Oliveira et al. [40], patients with a higher plasma homocysteine concentration show a faster progression of the disease by MSSS (Multiple Sclerosis Severity Score) and more pronounced disability according to EDSS (Expanded Disability Status Score). In addition, in this work it was revealed that, in patients with a higher concentration of homocysteine, there are increases in the concentration of tumor necrosis factor-alpha 1 receptor (TNF-α receptor 1) and ICAM. In the study by Guzel et al. [50], it was shown that the concentration of homocysteine is statistically significantly higher in patients with an EDSS greater than 5. The data obtained confirm the association of hyperhomocysteinemia with the progression and severity of disability in patients with MS.

Among the individual clinical manifestations of MS, of great interest is the association of hyperhomocysteinemia with cognitive impairments, in the development of which neurodegenerative changes and the atrophy of brain matter play a leading role. According to Fahmy et al. [48], the plasma homocysteine concentration is a statistically significant predictor of the development of cognitive impairment in MS. It was shown that the concentration of homocysteine statistically significantly negatively correlated with the total score on the Addenbrooke’s Cognitive Examination (ACE), as well as with the results of the auditory addition test at a given pace and the neuropsychological test for verbal associations for a given letter [48].

Important data on the relationship of hyperhomocysteinemia in MS with clinical and demographic characteristics were obtained in the study by Zoccolella et al. [51]. In this study, 217 patients with MS were examined, including 53 patients with the clinically isolated syndrome (CIS), 134 patients with remitting MS, 23 patients with secondary progressive MS, and 7 patients with primary progressive MS. In general, a small but statistically significant increase in the plasma homocysteine concentration was found in the group of patients with MS compared with healthy volunteers (*p* = 0.02). It is interesting to note that, in this work, it was shown that the concentration of homocysteine in MS is statistically significantly higher in men than in women (*p* ˂ 0.001), while in the control group there were no gender differences in this indicator. In addition, it was shown that the serum homocysteine concentration in CIS is statistically significantly lower than that in remitting and progressive MS (*p* = 0.04). Another factor associated with hyperhomocysteinemia, according to this work, is the duration of the disease; in patients with a disease duration of more than 22 months, the concentration of homocysteine was statistically significantly higher. The data obtained, indicating the association of hyperhomocysteinemia in MS with the male sex, the duration of the disease, and the presence of a reliable diagnosis, indirectly confirm the possible role of homocysteine in the development of neurodegenerative changes in MS [51].

The effect of the duration of MS on the homocysteine concentration was also shown in a study by Moghaddasi et al. [39], which included 75 patients with relapsing MS and 75 healthy volunteers with comparable ages. The patients with MS showed a statistically significant increase in the plasma homocysteine concentration, which correlated with the duration of the disease (r = 0.2; *p* = 0.05). In addition, this study showed that the concentration of homocysteine was statistically significantly higher in patients receiving interferon therapy (*p* = 0.01). The question of the effect of other MS medications on homocysteine levels needs further study.

To date, conflicting results have been obtained on the differences in plasma homocysteine concentrations in patients with various forms of MS. Several studies have shown that the homocysteine concentration does not statistically significantly differ in relapsing and progressive MS [37,49]. At the same time, other studies [41] have showed that the plasma homocysteine concentration in patients with progressive forms is statistically significantly higher than that in patients with relapsing-remitting MS, regardless of gender and age. The results of the main studies of homocysteine levels in patients with various forms of MS are presented in Table 1.

The effect of homocysteine on endothelial dysfunction was shown in vitro. It was reported that homocysteine (500 µM) decreases the viability and induces the apoptosis of human vascular endothelial cells. Increases in reactive oxygen species in endothelial cells treated with homocysteine were also found [70]. Increasing concentrations of reactive oxygen species in endothelial cells homocysteine may induce vascular inflammation [19,28]. The study by Barroso et al. showed the effect of the precursor of homocysteine S-adenosylhomocysteine on the expression of pro-inflammatory adhesion molecules such as ICAM-1, VCAM-1, and E-Selectin [71].

It is possible to suggest that homocysteine’s pro-inflammatory effects could be considered an additional mechanism of the participation of homocysteine in inflammatory diseases. In particular, the influence of homocysteine on MS pathogenesis could be mediated by not only affecting the BBB functioning but also by the modulatory effect of homocysteine on adaptive immune system cell function. Thus, Dawson et al. reported that homocysteine increases apoptotic death in resting T-cells in a dose-dependent manner (10–1000 µM) and modulates cytokine production by activation with anti-CD3 antibody peripheral blood mononuclear cells (PBMCs) or purified T-cells [72]. On the other hand, the ability of stimulated PBMCs and T-cells to produce homocysteine was shown [72,73].

**Figure 2:**
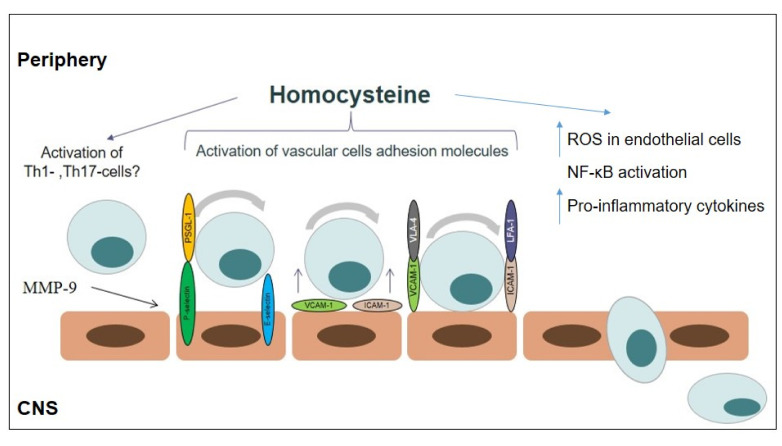
The possible mechanism action of homocysteine in multiple sclerosis pathogenesis: (i) oxidative stress; (ii) inflammation (upregulation of adhesion molecule expression, T-cell activation, inducing pro-inflammatory cytokine production).

The effect of homocysteine on CD4^+^-T-cells subsets was shown. According to Feng et al., homocysteine may induce Th1-cell proliferation and IFN-γ production [74]. The effect of homocysteine on Th17-cell differentiation and function also was shown [75,76]. In the study of Curro et al., the authors showed the stimulating effect of homocysteine on the mRNA expression of the Th17-differentiation cytokines IL-6 and IL-1β in human monocytes [77]. The effect of homocysteine on Th17-cells in vitro confirms the in vivo study. Thus, in the study by Fefelova et al., it was shown that the treatment of experimental rats with homocysteine (0.1 μM/g) increases the IL-17A and IFN-γ plasma levels [78]. Th1- and Th17-cells play a crucial role in MS pathogenesis. The ability of Th17-cells to disturb BBB by producing pro-inflammatory IL-17 and migrate into CNS by the expression of chemokine receptor CCR-6 (CD196) was demonstrated [79,80]. This suggests that homocysteine may indirectly affect the BBB functioning by activating the Th17-immune response. The possible mechanisms of action of homocysteine in MS pathogenesis are presented in Figure 2.

The homocysteine ability to induce B-cell proliferation in vitro and in vivo is also shown [81]. Overall, these data allow proposing the additional mechanism of the involvement of homocysteine in MS pathogenesis. However, the immunomodulatory effects of homocysteine in MS need to be further investigated.

## 4. Conclusions

Thus, hyperhomocysteinemia is an important factor in the pathogenesis of not only cardiovascular pathology but also in such an autoimmune disease of the nervous system as MS. One of the most important tasks now is searching for biomarkers that can predict the course of MS, which determines the treatment tactics [82]. Further study of the role of homocysteine and its precursors can help clarify the pathogenesis, identify the significance of an increase in homocysteine concentration on the course of MS, and develop therapeutic tactics for the treatment of MS.

## Figures and Tables

**Table 1:** Data of the main studies of homocysteine level in relapsing-remitting multiple sclerosis (RRMS), secondary-progressive multiple sclerosis (SPMS), primary-progressive multiple sclerosis (PPMS), and clinically isolated syndrome (CIS) patients.

Disease	Results of the Study	Authors
MS	No significant differences in the homocysteine serum levels between the MS group and healthy subjects.	Rio et al., 1994 [52]
MS	The increases in homocysteine in both the serum and CSF levels compared with healthy subjects.	Baig et al., 1995 [53]
RRMS SPMS PPMS	The increases in the homocysteine plasma levels in RRMS, SPMS, and PPMS patients (overall) compared with healthy subjects. No significant differences in the CSF homocysteine levels between the MS patients (overall) and the control group.	Vrethem et al., 2003 [54]
SPMS	The increases in the homocysteine plasma level in SPMS patients compared with healthy subjects.	Besler et al., 2003 [55]
RRMS SPMS	The increases in the homocysteine serum levels in RRMS and MS patients (overall) compared with healthy subjects.	Ashtari et al., 2005 [56]
RRMS SPMS PPMS	The increases in the homocysteine plasma levels in RRMS, SPMS, and PPMS patients compared with healthy subjects. No significant differences in the homocysteine plasma levels in MS patients, depending on the type of MS course.	Ramsaransing et al., 2006 [37]
RRMS	The increases in the homocysteine plasma levels in RRMS patients compared with healthy subjects.	Sahin et al., 2007 [57]
RRMS SPMS PPMS	The increases in the homocysteine plasma levels in RRMS, SPMS, and PPMS patients (overall) compared with healthy subjects. Association of a high homocysteine plasma level with cognitive impairment in MS patients (overall).	Russo et al., 2008 [47]
RRMS	The increases in the homocysteine plasma level in RRMS patients compared with healthy subjects.	Aksungar et al., 2008 [58]
RRMS	The increases in the homocysteine serum level in RRMS patients compared with healthy subjects. Association of a high homocysteine serum level with depression in RRMS patients.	Triantafyllou et al., 2008 [59]
RRMS SPMS PPMS	No significant differences in the homocysteine serum levels between the RRMS, SPMS, and PPMS groups (overall) and healthy subjects. No significant differences in the homocysteine plasma levels in MS patients, depending on the type of MS course. The increases in the homocysteine serum levels in male RRMS and SPMS patients compared with female RRMS and SPMS patients.	Teunissen et al., 2008 [49]
RRMS SPMS PPMS	No significant differences in the homocysteine serum levels between the RRMS, SPMS and PPMS groups (overall) and healthy subjects.	Kocer et al., 2009 [60]
RRMS SPMS	The increases in the homocysteine plasma levels in RRMS and SPMS patients (overall) compared with healthy subjects.	Salemi et al., 2010 [38]
RRMS SPMS PPMS	The increases in the homocysteine serum levels in RRMS, SPMS, and PPMS patients (overall) compared with the control group.	Zhu et al., 2011 [61]
RRMS SPMS PPMS CIS	The increases in the homocysteine plasma levels in RRMS, SPMS, PPMS, CIS patients (overall) compared with the control group. The increases in the homocysteine plasma level in male patients (overall) compared with female patients (overall). The decreases in the homocysteine plasma level in CIS patients compared with RRMS, SPMS, and PPMS patients. Association of a high homocysteine plasma level with the duration of disease.	Zoccolella et al., 2012 [51]
RRMS	The increases in the homocysteine plasma level in RRMS patients compared with healthy subjects. Association of a high homocysteine plasma level with the duration of disease and treatment with interferon in RRMS patients.	Moghaddasi et al., 2013 [39]
RRMS SPMS PPMS CIS	No significant differences in the homocysteine serum levels between the RRMS, SPMS, PPMS, and CIS patients (overall) and healthy subjects. No significant differences in the homocysteine serum levels between the RRMS and SPMS groups. No significant differences in the homocysteine serum levels between the treatment (with DMT) and non-treatment patients. The increases in the homocysteine serum level in male patients (overall) compared with female patients (overall).	Kararizou et al., 2013 [62]
RRMS	The increases in the homocysteine plasma level in males compared with females in both RRMS patients and healthy subjects.	Davis et al., 2014 [63]
RRMS RRMS (relapse) SPMS PPMS	The increases in the homocysteine plasma levels in RRMS patients during relapse compared with RRMS patients during remission and SPMS and PPMS patients. No significant differences in the homocysteine serum levels between the RRMS, SPMS, PPMS, and CIS patients (overall) and healthy subjects.	Adamczyk-Sowa et al., 2016 [64]
MS	No significant differences in the homocysteine serum levels between MS patients and healthy subjects. Association of a high homocysteine serum level with the cognitive impairments in MS patients.	Fahmy et al., 2018 [48]
RRMS SPMS PPMS	The increases in the homocysteine plasma levels in RRMS, SPMS, and PPMS patients (overall) compared with healthy subjects. The increases in the homocysteine plasma level in RRMS patients compared with SPMS and PPMS patients (overall). The increases in the ICAM-1 plasma levels in MS patients (overall) with higher homocysteine levels compared to the MS patients (overall) with lower homocysteine plasma levels.	Oliveira et al., 2018 [40]
MS	The increases in the homocysteine serum levels in RRMS patients during relapse compared with healthy subjects.	Pan et al., 2019 [65]
RRMS	The improvement with vitamin B12 and folic acid decreases in the homocysteine serum levels in RRMS patients.	Nozari et al., 2019 [66]
RRMS SPMS	The increases in the homocysteine serum levels in RRMS and SPMS patients compared with healthy subjects. Cladribine treatment decreases in homocysteine serum levels in SPMS patients.	Jamroz-Wiśniewska et al., 2020 [67]
RRMS SPMS PPMS	The increases in the homocysteine plasma levels in RRMS, SPMS, and PPMS patients (overall) with an EDSS score ≥3.	Flauzino et al., 2019 [68]
RRMS SPMS PPMS	The increases in the homocysteine serum levels in RRMS patients compared with healthy controls. No significant differences in the homocysteine serum levels between the SPMS and PPMS groups and healthy controls.	Li et al., 2020 [69]
